# Importance of magnetic resonance in the diagnosis of pleomorphic parotid adenoma. Clinical case series

**DOI:** 10.4317/medoral.27991

**Published:** 2026-03-07

**Authors:** José Paz-Expósito, Loreto Monsalve-Guil, Sonia Egido-Moreno, Álvaro Jiménez-Guerra, José López-López, Eugenio Velasco-Ortega, Iván Ortiz-Garcia

**Affiliations:** 1 Department of Radiology. Hospital Universitario Puerta del Mar. Cádiz, Spain; 2 Faculty of Dentistry. University of Seville.; 3 Department of Odontoestomatology. Faculty of Medicine and Health Sciences (School of Dentistry), University of Barcelona. University Campus of Bellvitge, Barcelona, Spain; 4 Dental Hospital University of Barcelona, (Barcelona University). Oral Health and Masticatory System Group (Bellvitge Biomedical Research Institute) IDIBELL, Barcelona, Spain

## Abstract

**Background:**

Parotid tumours account for approximately 3% of head and neck neoplasms, of which nearly 80% are benign. The most common being the Pleomorphic Adenoma (PA). Our objective was to analyze the morphological characteristics of the PA by Magnetic Resonance (MRI scan) and how it behaves in advanced diffusion (ADC-Apparent Diffusion Coefficient- value) and perfusion sequences.

**Material and Methods:**

Descriptive and inter-observer study of patients with a suspected PA diagnosis in an MRI scan and subsequent histological confirmation. All studies were carried out with the same MRI. The MRI protocol included enhanced sequences in T1 (T1W), T2 (T2W) diffusion study (DWI) and enhanced sequences in T1 with fat saturation (T1W FS) after injecting the contrast.

**Results:**

Of 113 patients that underwent magnetic resonance imaging due to a palpable nodule in the parotid region, a radiological diagnosis of Pleomorphic Adenoma (PA) was established in 56 patients. Thirteen were excluded due to a prior histopathological diagnosis, 39 of the 43 cases with suspected PA were confirmed (90.67%). They were morphologically well-defined homogeneous tumors, with an average value of high ADC (1.85x10-3mm2/s) and a type A perfusion curve (associated to benignity). The inter-observer concordance was 100%.

**Conclusions:**

PAs show typical morphological characteristics in an MRI scan. In case of diagnostic doubt, the diffusion and perfusion sequences help establish a definitive diagnosis.

## Introduction

Parotid tumours represent about 3% of all head and neck neoplasms (1). Approximately 80% are benign, the Pleomorphic Adenoma (PA) or mixed benign tumor being the most common for the parotid gland (2 , 3); while the most frequent malign tumor is the mucoepidermoid carcinoma (4 , 5). The PA affects most commonly middle aged women. It is a slow-growing tumor, typically unilocular and may remain asymptomatic for years (6 , 7). This slow and asymptomatic nature of its growth often leads to a large tumor size by the time of diagnosis. Usually, signs such as rapid growth, the appearance of local symptoms (like pain, facial paralysis and ulceration) and the existence of enlarged lymph nodes indicate the possibility of malignancy. Treatment of PA is surgical, non the less, this surgery may not be as aggressive as it is for malign tumors, and be restricted to a partial parotidectomy or local excision of the tumor, usually with conservation of the facial nerve (1 , 8).

Imaging techniques play an important role in the diagnostic process to facilitate appropriate surgical planning. Despite the availability of various imaging techniques, such as ultrasound and CT, magnetic resonance imaging (MRI) provides superior anatomical definition and more precise delineation of lesion margins, outperforming other modalities and allowing assessment of perineural and intracranial tumor extension. The introduction of advanced MRI sequences such as diffusion sequences (DWI) and the perfusion curve after contrast is added have resulted in higher diagnostic accuracy in conventional diagnosis sequences of pleomorphic adenoma (9 - 10).

Qualitative MRI features combined with quantitative ADC values offer a non-invasive, accurate approach for diagnosing PA (11). A retrospective study including patients with parotid masses that underwent surgical resection and preoperative MRI. Patient and MRI characteristics, including quantitative apparent diffusion coefficient (ADC) values, were analyzed to identify predictors of PA. Among 157 patients, 86 (55%) had PA. MRI sensitivity and specificity for PA was 56% and 96%, respectively. Key predictors of PA included higher ADC values, T2 hyperintense signal, lobulated tumor contour, and absence of dumbbell shape (11).

MRI - diffusion-weighted imaging findings and ADC also contribute to the diagnosis of parotid tumors (12). Patients with parotid masses diagnosed using histopathology and/or cytology were enrolled in this retrospective study. MRI findings and ADC values were compared between benign-malignant groups and pleomorphic adenoma vs Warth-in's tumor groups. Sixty tumors (48 benign, 12 malignant) were evaluated in a total of 60 patients. The mean lesion size was 26 (±10,11-61)mm. T2 dominantly hyperintense/with hypointensity signal was seen in 87% of pleomorphic adenomas and T2 dominantly hypointense / with hyperintesity signal was encountered in 64% of all Warthin's tumors. Seven (28%) Warthin's tumors were misdiagnosed as pleomorphic adenomas and two others (8%) as malignant tumors. The commonly used mean ADC value was 1.6±0.6×10-3mm2 s-1 for benign tumors, 0.8±0.3×10-3mm2 s-1 for malign tumors, 1 (0.9-1.8)×10-3mm2 s-1 for Warthin's tumors, and 1.9±0.3×10-3mm2 s-1 for pleomorphic adenomas. In addition to benign-malignant differentiation, the added ADC measurement may also be useful for differentiating Warthin's tumors from pleomorphic adenomas (12).

The aim of this study was to investigate the relationship between histopathological characteristics of PA and MRI - diffusion-weighted imaging findings and value the contribution of apparent diffusion coefficient (ADC) to the diagnosis.

## Material and Methods

This retrospective study, which was approved by the Ethics Committee of Universitary Hospital Puerta del Mar of Faculty of Medicine of Cádiz. The inclusion criteria applied to patients over the age of 18, who had undergone a parotid MRI between January 2016 and March 2025, with radiological suspicion of PA.

All studies were carried out with the same MRI (Philips Healthcare 1.5 Tesla). The MRI protocol included enhanced sequences in T1 (T1W), T2 (T2W) diffusion study (DWI) and enhanced sequences in T1 with fat saturation (T1W FS) after injecting the contrast. The contrast used was gadolinium (1 mmol/ml in injectable solution, with a dosage of 0.2 ml/kg of weight) administered with an injection pump that made a total of 8 measurements (every 30 seconds). Demographic variables (age and gender) were collected and analyzed, as well as the clinical features and the type of surgical treatment given.

A radiologist (JPE with 10 years of experience in head and neck MRI) and an expert in oral pathology (JLL with over 20 years of experience in radiology and oral pathology) did an independent assessment of the parotid MRI images, masking both the radiological case report and the histopathological results. The following variables were recorded:

- Precontrast study: Location (superficial, or deep lobule, or both), number of lesions; morphology (oval, lobulated or irregular); signal intensity (homogeneous or heterogeneous); margins (well defined, not well defined or spiculated); capsule presence (complete or incomplete), extra-glandular invasion and the presence of lymphadenopathy.

- Diffusion sequence (DWI): The Apparent Diffusion Coefficient (ADC) indicates the cellular degree of the tumor. A low ADC value indicates high cellularity.

- Post-contrast sequences: Curves A, B and D are related to benignity while C (maximum contrast before 120'' and washout higher than 30%) is related to malignancy.

The diagnosis of radiological suspicion was verified with the histopathological analysis, thus being the standard reference.

The statistical analysis of the numerical data was performed with the SPSS v 24.0 programme (IBM Corp). The average and the range of continuous quantitative variables and the percentage of the frequencies of other variables were calculated.

## Results

During the study period, parotid MRI was performed on 113 patients, all of whom presented with signs and symptoms of a palpable nodule. The radiological diagnosis of PA was established in 56 patients. 13 of the 56 cases were excluded: One due to the existence of a prior histological diagnosis of PA obtained by fine needle aspiration puncture (PAAF) of the lesion, which interfered with the study, and 12 cases more were discarded due to no histological confirmation being available or not require surgery treatment. Of the 43 operated patients, the PA diagnosis was confirmed in 39 cases, with the histopathological study of removed surgical piece (90.1%). Basal cell adenoma was the histopathological result for the 4 cases with no concordance.

The average age of the 39 confirmed PA cases was 43 years (22-62 years) 17 of which were women and 22 men. All patients had only one type of lesion, seventeen on the left side of the parotid gland and twenty-two on the right. Twenty-six (66.7%) of them affected the superficial lobe, six the deep lobe and seven affected both. Table 1 summarizes the radiological characteristics of the series with the number of cases in each one.

**Figure 1:**
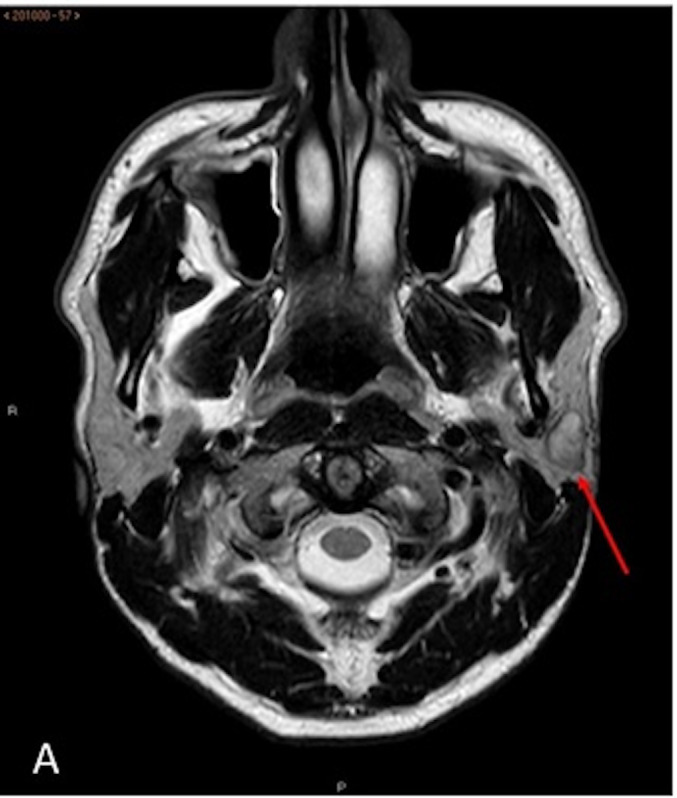
Basal Magnetic Resonance. Turbo Spin Echo Sequence T2 (TSE, T2) on axial plane. A well-defined and partially encapsulated left parotid ovoid tumor can be seen (red arrow) at the retromandibular level on the superficial lobule, showing a discreetly heterogeneous signal.

Table 1Morphologically, these were oval or lobulated contour lesions all encapsulated within well-defined margins. More than half (23 of 39) had homogeneous signal strength. The other sixteen had a slightly heterogeneous signal strength, indicating a mixed component (Figure 1). None of them presented extra capsular invasion data, signs of infiltration of neither the facial nerve nor lymphadenopathies suspected of cervical chain malignancy.

1

**Figure 2:**
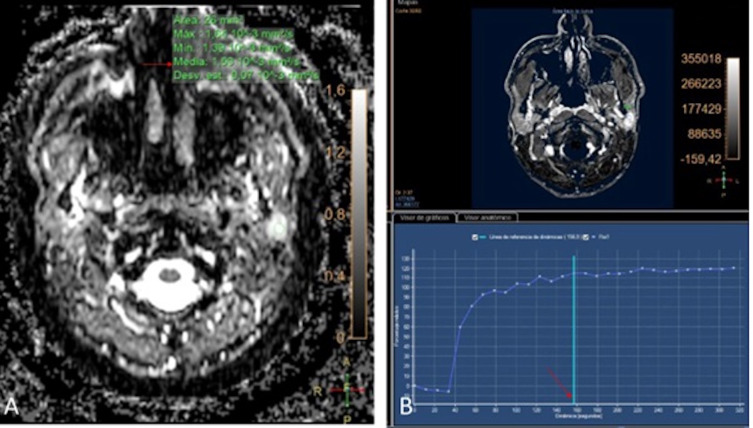
Image A: In the diffusion sequence, the left parotid tumor does not restrict and the ADC coefficient calculation corresponds to 1.53 (x10-3mm2 /s) (red arrow). Image B: In the perfusion sequence, the tumor presents a contrast capture with a retarded peak curve at 156.9'’ (red arrow) with progressive enhancement and no washing (type A curve).

In the DWI sequence, the nodules presented an average ADC value of 1.85x10-3mm2/s (1.26-2.59) (Figure 2, image A). After the administration of the contrast, all the lesions presented a progressive enhancement reaching 120" with a maximum setting and with no subsequent washing (type A curve, with benign behavior) (Figure 2, image B). The interobserver concordance was 100% in the final PA diagnosis.

2

Surgical treatment was given in all of the cases. Ten patients with full parotidectomy, while 29 preferred conservative surgery with lumpectomy (n=8) or partial parotidectomy (n=10).

## Discussion

The PA is the most frequent of either major or minor salivary gland tumors and it is the most frequent parotid gland tumor (13 , 14). Due to its benign behavior, it is very important to make a correct preoperative diagnosis, to allow consideration of all surgical options or to opt for monitoring in patients with surgical risks. PAs have been morphologically described as masses with well-defined borders surrounded by a complete or incomplete capsule, without infiltration into neighboring structures and with homogeneous signal intensity (15 , 16). Among our patients, 23 lesions had completely benign criteria, while 16 had a heterogeneous signal intensity, which could create doubts regarding the PA diagnosis. It should be remembered that heterogeneous enhancement, along with other parameters such as a low apparent diffusion coefficient or a high signal intensity ratio on T1-weighted images with gadolinium and fat suppression, are signs of a worse prognosis in parotid gland neoplasms (17).

The PA in DWI sequences will have a high ADC due to its low cellularity. One of the first original works that studied the behavior of PA in diffusion sequences was the one done by Yabuuchi et al. (9) where they established an ADC diagnostic value for PA&gt;1.4x10-3mm2/s. In a later article, Matsusue et al. (18) established the same ADC threshold value for the PA diagnosis. Both are retrospective studies. In our series, 79.5% of the lesions (31 of 39) had an ADC1.40x10-3 mm2/s. The eight cases that were below this value had an ADC between 1.3-1.39x10-3mm2/s, which was still above the threshold value established by both authors (10 , 19 , 20) for the Warthin tumor and those malign lesions which ADC set at &lt;1x10-3mm2/s. These eight lesions morphologically corresponded to the most heterogeneous lesions. The only work published on this subject with prospective recruitment is the one carried out by Milad et al. Only 8 of the 46 parotid tumors analyzed were PAs. They established an ADC value of 1.67x10-3mm2/s, despite it being the average for all the benign lesions (including cysts and adenopathies), not only for PAs. However, nineteen (48.7%) of our cases were also above this threshold level. Our work is the only prospective design, which exclusively studies PA behaviour.

The use of contrast in the diagnosis of parotid lesions has also been the object of a number of investigations (9 , 17 - 22). All agree that benign lesions have a homogeneous enhancement. The perfusion curve morphology is also a characteristic Curves A, B and D that indicate benignancy, curve A being more indicative of PA. All our cases showed a type A enhancement curve. Some authors such as Stefanovic et al. (20), reached the conclusion that the type of curve is decisive for the correct relation of the lesions in specific cases that had an ADC between 1.2 and 1.4x10-3mm2/s. Yabuuchi et al. (9) studied this same aspect, coming to the conclusion that in lesions that showed washing (types B and C), resorting to the interpretation of the diffusion study is required, whilst the A and D curves correspond to diagnoses of benign lesions. Following the protocols that interpret all of the advanced MRI sequences together, all our lesions were conclusively PAs.

Four cases from the 43 initially reviewed, which radiologically appeared to be PA, were finally diagnosed as monomorphic or basal cell adenoma, with a concordance greater than 90.6%. This kind of tumor is not very frequent; being also a benign type (22 , 23). The main difference with the PA is the higher recurrence rate. The radiological findings are indistinguishable from the PA. Due to the low prevalence, there are no studies related to the behavior of these tumors neither in advanced MRI sequences or about the existence of any difference in behavior with the PAs.

MRI is a very effective imaging method in the diagnosis of PA, which has been described for years (24 - 26). Advances in functional MRI imaging, specifically diffusion-weighted and dynamic contrast-enhanced or perfusion sequences, can also help differentiate between benign and malignant tumors by providing information on tumor microcirculation and microcellularity. Morphological MRI allows for the precise identification of various findings of great interest in distinguishing between benign and malignant tumors, such as location (superficial and/or deep lobe), borders (well-defined or poorly defined), infiltration of fat or adjacent spaces, T1- and T2-weighted signal characteristics, oval or multilobulated appearance, the presence or absence of perineural infiltration (facial or mandibular nerve), and lymphadenopathy. Furthermore, functional MRI using diffusion-weighted sequences allows for the determination of the apparent diffusion coefficient (ADC) and the type of uptake curve in dynamic contrast-enhanced or perfusion sequences (11 , 12). The combination of morphological and functional imaging allowed a preoperative diagnosis to be established in 90.7% of the patients in our series. MRI is the first-choice technique for the evaluation of PA, having surpassed other diagnostic imaging methods, such as ultrasound or CT, in the diagnosis of salivary tumor pathology. MRI helps in the differential diagnosis of PA with other parotid tumors, benign and malignant, by means of the morphological and fundamentally functional image, as it presents different ADC values and perfusion curves (27 - 29). MRI imaging technique has improved in recent years not only for diagnosis but also for treatment planning, through 3D reconstruction and the possibility to fuse images with computed tomography (CT) (30).

The main limitation of this study is that it was designed as a diagnostic and follow-up case series study, rather than a blinded clinical trial. Furthermore, only cases with suspected pleomorphic adenoma after MRI were evaluated.

Our study is restricted to studying the behavior of PAs only, without comparing them with other types of tumors, such as the Warthin tumor or malign types (mucoepidermoid or metastasis among others). The next step will be to compare the behavior of our series of PAs with other tumors, but we need the analysis of more cases to obtain statistically relevant differences. This is also the only study among observers with a varied range of experience (one expert in parotid MRI and another expert in oral pathology), that reached 100% agreement. This fact shows that the PA diagnosis is easy with basic knowledge of morphology and behavior in the diffusion study including contrast.

## Conclusions

Pleomorphic Adenoma are the most common benign tumors in the parotid gland. They are morphologically well-defined lesions, without infiltration into neighboring, homogeneous structures. Advanced MRI sequences play a crucial role in imaging and in the diagnosis of these lesions. The lack of washing after administration of contrast and a &gt;1.4x10-3mm2/s ADC is diagnostic of PA.

## Figures and Tables

**Table 1:** Table Characteristics of Pleomorphic Adenomas in Magnetic Resonance. Of the 43 cases operated on with diagnosis by AP MRI, 39 were coincidences with diagnostic suspicion and 4 turned out to be Basal cell adenoma (0.061% concordance).

Variable	Characteristics	Number of cases (%)
Location	Superficial lobeDeep lobeBoth lobes	26 (66,7)6 (15,4)7 (17,9)
Morphology	OvalLobed	17 (43,6)22 (56,4)
Margins	Well definedPoorly defined	37 (94,9)2 (5,1%)
Capsule	CompleteIncomplete	32 (82,1)7 (17,9)
Signal intensity	HomogeneousHeterogeneous	23 (59)16 (41)
Diffusion sequence: ADC range (x10-3mm2/s)	<1.41.4-1.59>1.6	8 (20,5)12 (30,8)19 (48,7)
Perfusion curve	Type A	39 (100)

1

## Data Availability

Declared none.
